# Proceedings of the Society of Homœopathicians

**Published:** 1899-01

**Authors:** 


### PROCEEDINGS OF THE SOCIETY OF HOMŒOPATHICIANS.

Dr. F. W. Patch presented the following resolution to the meeting:

WHEREAS, We believe the law of similars to be the law of cure, and the similarity of symptoms to be the only guide in the administration of the law; therefore be it

*Resolved*, That we discountenance the practice known as the "Antidotal Method," as such, because it is opposed to the foregoing principles and to the tenets of this society.

### DISCUSSION OF RESOLUTION BROUGHT FORWARD BY DR. PATCH IN RE THE SO-CALLED ANTIDOTAL TREATMENT.

Dr. Pease—This resolution causes me a great deal of surprise. Considering the relation that an absent member of this Association bears to this resolution, it seems to me, where such an important matter is concerned, that absent member should have an opportunity to discuss it, because I fear that if the resolution should be adopted this evening there would be more misunderstanding and misinterpretation of Dr. Patch's honest endeavor to do right than there has been of other aims of this Association. I presume every member here, when speaking of the Society of Homœopathicians to homœopathic physicians who are not friendly toward the Association, finds it as hard to bear with the views of these physicians, patiently, as it is with objections to the so-called antidotal system of treatment.

The resolution seems to utterly throw out of consideration the question as to what is included under the title of antidotal treatment. Members of this Association are very much interested in the matter, both from having given considerable time to experimental investigation, and in reporting cases that have substantiated the treatment. The adoption of this resolution might seem strange to some of our absent members.

Dr. Davis—I think there is not a member of this Society, present or absent, who recognizes more than one law of cure. I do not believe that publishing some cures that have been made has led any one to suppose that there are two methods or laws of cure. I believe that no remedy can cure except in accordance with the homœopathic law, but we may use remedies empirically, although they may be potentized, and we may select them without any question as to whether we are doing it according to the requirements of that law or not. I want to ask the question, Are we then practicing Homœopathy? We may say that we practice Homœopathy by mistake sometimes, but we are not the physicians who do things by mistake. That is not what we come here for. We must not do things slip-shod or by chance, and without putting into practice the laws of homœopathic practice. It is for this reason that I have favored the bringing forward of this resolution, not because I would throw out anybody's influence or throw cold water on anybody's practice if they practice according to their conscience, but because I would not care to have my influence govern this Society. I should not feel at all hurt if the Society, as a Society, rejected my position upon any question. It is a fact that the practice known as the "antidotal method" is the stumbling block of this Society, because it is supposed to be something different from Hahnemann's method, which uses proved remedies only. That method requires that even the so-called nosodes should first be proved. We want to clear our Society of all such reflections. We wish to set the position of the Society right in regard to every method of cure, mental science, Christian science, and everything else. If there is anything in what is called the antidotal method, we cannot get away from our law of cure.

Dr. Adams—I would ask as a favor that in any further discussion of this Society, we do not use the word "antidotal." We call it "antidotal" unjustly, and then damn it.

Dr. Kimball—There is no question but that the Society is looked upon as introducing a method which is different from the Law of Similars, as exemplified by Hahnemann, and if the Society is to live as a Society it must put itself on record as opposed to the "antidotal method." It is simply to put the Society on record as opposed to anything that is not in accordance with the Law of Similars. It is really necessary for the Society to take some stand in the matter, and I do not see how anybody can oppose a resolution which endorses the Law of Similars.

Dr. Adams—I believe we should put ourselves right with outsiders in regard to this matter.

Dr. Kimball—It is simply to put the Society right, and put it right with the public as well.

Dr. Adams—This Society should adopt and stand by the truth, even if the whole world reject it.

Dr. Close—Let us keep in mind the purpose of this resolution. If the views expressed in the Society meetings have been misunderstood, and it seems wise to take steps to remove misapprehensions, we can do so. How shall we express what we desire to accomplish?

Dr. Patch—I have a jealous feeling as far as the standing of our Society is concerned with outsiders. We want the world to know that it stands for truth. As far as any use of remedies is concerned, we certainly want to cling to what is right, to what Hahnemann said. We follow no method aside from the method of pure Homœopathy, and if anything can be added to the resolution to make it stronger for the right, let it be so. I, personally, want to make it clear that there is no ill-will in this resolution.

Dr. Pease—Of course there is no question about the ill-will. I do not know anybody who holds the good of the Society more closely at heart than Dr. Sawyer or myself. Let us think about this and make no mistake. Is it necessary that this should be adopted to-night? At present it is not in accordance with my belief.

Dr. Close—I think it would be wise to further consider the matter, and in the endeavor to formulate our thought, to include in the resolution a brief statement as to the source of the misunderstanding that is abroad. Shall it be delegated to a committee for consideration and discussion as to the best form in which to present the subject for our action? The resolution does not clearly set forth the grounds for such action.

Dr. Kennedy—I believe that we ought to give ourselves time to think it over, and our Brother Adams, who has not had time to consider it, should have time to think it over. Personally, I believe that Dr. Adams, Dr. Pease, and Dr. Sawyer have the interests of Homœopathy at heart. I am sorry that Dr. Sawyer is not here. I told Dr. Pease to-day, in talking with him in regard to this very proposition, that I thought it would be presented some time during the session. I told Dr. Pease, and he seemed to take it kindly, how I felt, and why I felt as I did. I told him how, in our section of the country, particularly, where quite a number of the members of this Society work, we are hearing slurs against our Society. We are called the Society that follows the "antidotal method." Now the purpose of this resolution is to simply put ourselves on record definitely, in order that these parties outside can no longer, with any show of truth, say anything of this kind. It is needless to repeat what has already been said, that there is no personal feeling with regard to any one member of the Society by any one of us. We, as an Association, do not feel that we should be labeled as being governed by this method. I supposed that Dr. Adams, in common with other members, had been made acquainted with the existing state of things. I do not wonder that he should feel for the moment that we are attempting to "spring it on him," but I do not think, Mr. Chairman, we had any such idea. I think it is well to take it deliberately and carefully because we are united in this cause for which the Society was formed, but I do not wish, and I do not think there is a single member who wishes that our Society should be committed to any one individual opinion with regard to any one phase of treatment. I believe that in different form the resolution may be presented and accepted, as I have no doubt it will be when fully understood and discussed. We will still feel that as individuals, we can hold individual opinions. If I did not feel that I could hold an individual opinion differing from others, I should not wish to come to the meeting. I think we will be able to hold individual views, and it will be perfectly proper to hold such views, but that no one of us will ask or expect that the Society, as such, shall be governed by, or be said to be governed by (with any degree of truth) the opinion of any individual member. I think, also, it is well to put on record what we say. I favor granting any time that is reasonable for the members to think this over.

Dr. Kimball—If the members wish it referred to a committee I will withdraw my former motion, and move that it be referred to a committee of three, which shall include the Chairman.

Motion carried.

The Chairman appointed Drs. Patch, Pease, and Close. The Committee withdrew and reported the following resolution:

WHEREAS, We believe the Law of Similars to be the law of cure, and similarity of symptoms to be the only guide in the selection of the curative remedy; and

WHEREAS, The treatment of artificial or drug diseases, the same as natural diseases, must be governed by homœopathic principles; therefore be it

*Resolved*, That the empirical prescription of remedies for any purpose whatever, whether under the name of "antidotal treatment" or any other name, has not been, and cannot be endorsed by this Society.

*Resolved*, That this Society repudiates the practice of prescribing any drug, as an "antidote," irrespective of the actual symptoms of the case, whether it be to give a high potency of a drug known to have been used in crude form, or whether it be to give a dose of Sulphur "when well-selected remedies fail to act."

Resolution adopted.

